# A Nomogram Based on Clinicopathological and Ultrasound Imaging Characteristics for Predicting Cervical Lymph Node Metastasis in cN0 Unilateral Papillary Thyroid Microcarcinoma

**DOI:** 10.3389/fsurg.2021.742328

**Published:** 2021-12-03

**Authors:** Lina Zhang, Yuwei Ling, Ye Zhao, Kaifu Li, Jing Zhao, Hua Kang

**Affiliations:** ^1^Department of General Surgery, Center for Thyroid and Breast Surgery, Xuanwu Hospital, Capital Medical University, Beijing, China; ^2^Department of Colorectal Surgery, PLA Rocket Force Characteristics Medical Center, Beijing, China

**Keywords:** papillary thyroid microcarcinoma, unilateral, cervical lymph node metastasis, ultrasound imaging, nomogram

## Abstract

**Objective:** The aim of this study was to establish a practical nomogram for preoperatively predicting the possibility of cervical lymph node metastasis (CLNM) based on clinicopathological and ultrasound (US) imaging characteristics in patients with clinically node-negative (cN0) unilateral papillary thyroid microcarcinoma (PTMC) in order to determine a personal surgical volume and therapeutic strategy.

**Methods:** A total of 269 consecutive patients diagnosed with cN0 unilateral PTMC by postoperative pathological examination from January 2018 to December 2020 were retrospectively analyzed. All the patients underwent lobectomy or thyroidectomy with routine prophylactic central lymph node dissection (CLND) and were divided into a CLNM group and a non-CLNM group. Using logistic regression, the least absolute shrinkage and selection operator (LASSO) regression analysis was applied to determine the risk factors for CLNM in patients with unilateral cN0 PTMC. A nomogram including risk-factor screening using LASSO regression for predicting the CLNM in patients with cN0 unilateral PTMC was further developed and validated.

**Results:** Risk factors identified by LASSO regression, including age, sex, tumor size, presence of extrathyroidal extension (ETE), tumor diameter/lobe thickness (D/T), tumor location, and coexistent benign lesions, were potential predictors for CLNM in patients with cN0 unilateral PTMC. Meanwhile, age (odds ratio [OR] = 0.261, 95% CI.104–0.605; *P* = 0.003), sex (men: OR = 3.866; 95% CI 1.758–8.880; *P* < 0.001), ETE (OR = 3.821; 95% CI 1.168–13.861; *P* = 0.032), D/T (OR = 72.411; 95% CI 5.483–1212.497; *P* < 0.001), and coexistent benign lesions (OR = 3.112 95% CI 1.407–7.303; *P* = 0.007) were shown to be significantly related to CLNM by multivariant logistic regression. A nomogram for predicting CLNM in patients with cN0 unilateral PTMC was established based on the risk factors identified by the LASSO regression analysis. The receiver operating characteristic (ROC) curve for predicting CLNM by nomogram showed that the area under the curve (AUC) was 0.777 and exhibited an excellent consistency.

**Conclusions:** A nomogram based on clinical and US imaging characteristics for predicting the probability of CLNM in patients with cN0 unilateral PTMC was developed, which showed a favorable predictive value and consistency. Further prospective research to observe the oncological outcomes is necessary to determine whether the nomogram could potentially guide a personalized surgical volume and surgical approach.

## Introduction

Papillary thyroid microcarcinoma (PTMC) is defined as papillary thyroid carcinoma (PTC) with a tumor of maximum diameter ≤ 10 mm, and this subtype constitutes 30% of PTC cases (1, 2). PTMC accounts for 3% of the deaths of thyroid cancer and 5% of the deaths of PTC. Although PTMC is characterized as a kind of indolent tumor, the presence of lymph node metastasis always relates to unfavorable oncological outcomes. According to previous studies, about 25% to 64% of patients with PTMC have cervical lymph node metastasis (CLNM) (3), especially in the central compartment (4, 5). Meanwhile, PTMC patients with CLNM exhibit a high recurrence rate compared to those without CLNM (3). Whether it is necessary to perform routine prophylactic central lymph node dissection (CLND) for patients without clinical evidence of CLNM, especially for those with unilateral lesions, remains controversial (6). The 2015 American Thyroid Association (ATA) management guidelines for PTC do not recommend routine prophylactic CLND for T1 or T2, noninvasive, and clinically node-negative PTC (cN0) (1). The Chinese and Japanese guidelines for PTC recommend that prophylactic CLND should be performed with proper protection of the parathyroid gland and recurrent laryngeal nerve (RLN) (7, 8). Undoubtedly, unnecessary CLND will increase the incidence of hypoparathyroidism and RLN injury. Accordingly, accurate assessment of the status of cervical lymph nodes is crucial.

Although preoperative, high-resolution ultrasound (HUS) plays an important role in CLNM evaluation, it has a fairly low sensitivity (9). CLNMs are found in 33.2–50% of patients with PTMC, including patients preoperatively diagnosed with cN0 PTMC (10–12). Therefore, it is important to develop a novel method for accurately evaluating the status of cervical lymph nodes, to provide guidance for a personalized surgical volume and surgical approach. In the published literature, several researchers have proposed potential clinicopathological risk factors for CLNM and developed models for the preoperative evaluation of CLNM in patients with PTMC. Sun et al. (13) developed a nomogram based on ultrasound (US) features and clinical characteristics for the preoperative diagnosis of CLNM for PTMC. The study of Chen et al. (14) constructed a risk prediction model based on US imaging features and BRAF^V600E^ mutation. However, they have not provided further verification of the results of their studies, which limits the accuracy and credibility of the results. Thus, our study aimed to establish a practical nomogram for preoperatively predicting the possibility of CLNM based on clinicopathological and US imaging characteristics in order to determine a personal surgical volume and therapeutic strategy.

## Materials and Methods

### Patients

Consecutive patients who were admitted to the Center for Thyroid and Breast Surgery, Department of General Surgery, Xuanwu Hospital, from January 2018 to December 2020 and who were diagnosed with PTMC by postoperative pathological examination were retrospectively analyzed. The inclusion criteria were as follows: (a) no abnormally enlarged lymph node was detected by Ultrasound (US), physical examination, or other imaging examination approaches; (b) lobectomy or thyroidectomy with routine prophylactic CLND was performed; (c) the maximum tumor diameter was ≤ 10 mm on US; (d) tumors were located in the unilateral thyroid lobe; and (e) comprehensive clinicopathological and US imaging features were recorded. We excluded patients due to the following reasons: (a) the maximum tumor diameter was > 10 mm on US; (b) tumors were located in the bilateral thyroid lobe or the thyroid isthmus; (c) a diagnosis of thyroid benign disease was made preoperatively by fine-needle aspiration (FNA); (d) enlarged lymph nodes were detected by US, physical examination, or other imaging examination approaches and verified by FNA as clinically positive nodes (cN+); (e) the US image was absent or insufficient; (f) there was a history of neck radiation; (g) there were complications with other head and neck malignant tumors; and (h) reoperation was performed.

### US Imaging

**Figure 1:**
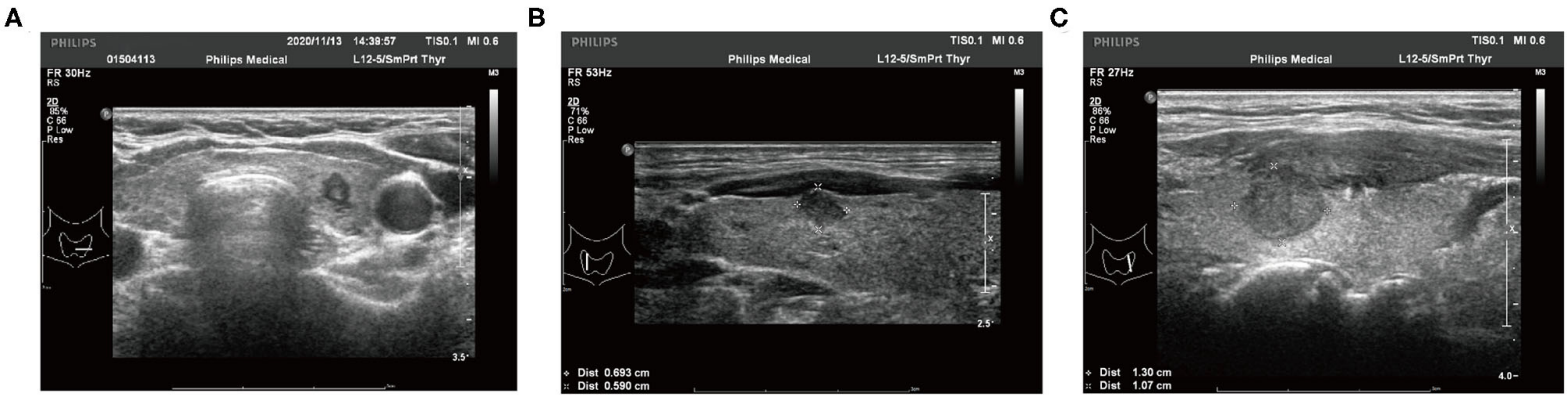
Typical ultrasound imaging of different extrathyroidal extension status. **(A)** The tumor is totally localized in the thyroid lobe. **(B)** The tumor invades the capsule of the thyroid lobe. **(C)** The tumor presents as an extrathyroidal extension.

Two experienced radiologists independently recorded and reviewed all preoperative US features using standardized institutional protocols. If the radiologists disagreed on a conclusion, the final decision was decided between them after a discussion. The following imaging features of each malignant lesion were analyzed: tumor size (the maximum diameter of the primary tumor), margin, shape, multifocality, aspect ratio (height divided by width on transverse views, A/T), microcalcification, internal echo pattern, extrathyroidal extension (ETE), and thickness of the thyroid lobe. The thickness of the thyroid lobe refers to the anteroposterior diameter of the thyroid gland measured by US. The measurement position should be selected on the edge of the hyperechoic line of the capsule at the thickest part of the thyroid under light pressure. The ratio of tumor maximum diameter to ipsilateral thyroid lobe thickness was identified as the diameter/thickness (D/T). The typical US imaging of different ETE statuses is shown in Figure 1.

### Logistic Regression and Least Absolute Shrinkage and Selection Operator Regression Analysis

The enrolled patients were first divided into a training set (2019–2020 patients) and a validation set (2018 patients). In order to discover the risk factors for lymph node metastasis in patients with cN0 unilateral PTMC, univariate logistic regression analysis was performed using the R Stats Package, including analysis of the potential risk factors based on clinicopathological and US imaging features. After screening by univariable logistic regression analysis, factors with statistical significance (*P* < 0.10) were identified as potential risk factors. The R package “glmnet” version 4.1-1 was used to perform the least absolute shrinkage and selection operator (LASSO) regression analysis. The 10-fold cross-validation was applied in the LASSO regression analysis to determine the optimal penalty parameter (λ), and dimension reduction was performed on the potential risk factors to reduce the interference or redundant factors in order to select the primary predictive factors and build a relatively refined model. Factors after screening by LASSO regression analysis were included in the multivariate logistic regression analysis to further determine the risk factors for CLNM in patients with PTMC.

### Development and Validation of the Nomogram

The R package “rms” version 6.1 was applied to construct the nomogram, including risk-factor screening using LASSO regression for predicting the CLNM in patients with cN0 unilateral PTMC. The length of the line corresponding to each factor in the nomogram reflected the contribution of each factor to lymph node metastasis in PTMC. The risk score was calculated by the R package “nomogramFormula” version 1.2. The prediction value of CLNM by the nomogram was examined using calibration curves.

### Statistical Analysis

Data were analyzed with the R software version 4.0.3. Receiver operating characteristic (ROC) curves were used to evaluate the predictive value of the nomogram and determine the cutoff values. Thresholds with the highest sensitivity–specificity sum were calculated using the R package “pROC” version 1.17.0.1 and plotted in the ROC curve. A *P* < 0.05 was considered statistically significant.

### Ethics Approval

The study was approved by the Ethics Committee of Xuanwu Hospital, Capital Medical University, based in Beijing, China (April 20, 2020; ID [2020]055). Participant data were kept confidential, and the details are presented such that the identity of the participants cannot be ascertained. The requirement for informed consent was waived because of the retrospective nature of the study. All procedures performed in this study involving human participants were conducted following the ethical standards of the institutional research committees and the Declaration of Helsinki (as revised in 2013).

## Results

### General Characteristics

A total of 392 consecutive patients diagnosed with PTMC were retrospectively analyzed. Among the 392 cases, postoperative pathological examination reports indicated that tumors were located in the bilateral thyroid lobe or isthmus in 70 cases; central or lateral lymph node metastases were detected by the preoperative US and confirmed by FNA (cN1) in 16 cases; PTMC was accidentally discovered postoperatively in four cases following a diagnosis of thyroid benign disease preoperatively by FNA; the maximum tumor diameter measured by US was >10 mm in 31 cases. Eventually, 269 participants (including 54 male and 126 female) were included in the study. The training set included 180 cases and the validation set included 89 cases. According to the postoperative pathological examination reports, the overall incidence of CLNM metastasis was 36.1% (97/269).

### The Risk Factors of Lymph Node Metastasis in Patients With cN0 Unilateral PTMC

**Figure 2:**
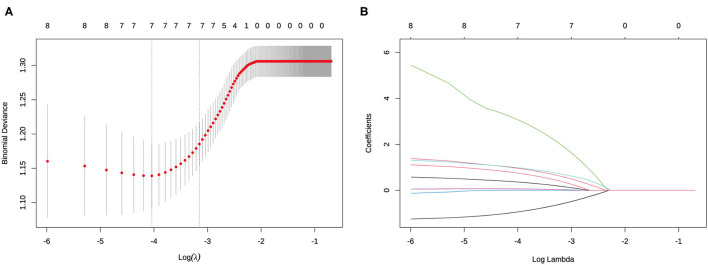
Determination of the risk factors of cervical lymph node metastasis (CLNM) in clinically node-negative (cN0) unilateral papillary thyroid microcarcinoma (PTMC) patients by least absolute shrinkage and selection operator (LASSO) regression analysis. **(A)** LASSO regression analysis of the risk factors identified by univariant logistic regression analysis. **(B)** Coefficients of the risk factors in LASSO regression analysis.

Initially, the result of the univariate logistic regression analysis based on the training set demonstrated that age (odds ratio [OR] = 0.352, 95% CI.16–0.726; *P* = 0.006), sex (men: OR = 2.405; 95% CI 1.249–4.665; *P* < 0.001), tumor size (OR = 1.253; 95% CI 1.055–1.499; *P* = 0.012), presence of ETE (OR = 5.519; 95% CI 1.934–17.966; *P* < 0.001), D/T (OR = 19.985, 95% CI 2.391–186.932; *P* = 0.007), location (middle: OR = 1.648, 95% CI 0.591–5.371, *P* = 0.366; lower: OR = 2.782, 95% CI.959–9.35, *P* = 0.073), and coexistent benign lesions (OR = 1.817; 95% CI.943–3.608, *P* = 0.08) were associated with CLNM in cN0 unilateral PTMC (Table 1). Then, in order to avoid the influence of confounding factors, LASSO regression analysis showed the above seven factors had non-zero coefficients, which indicated that the age, sex, tumor size, presence of ETE, D/T, tumor location, and coexistent benign lesions were potential predictors of the prediction model (Figure 2). Finally, the result of multivariate logistic regression analysis revealed that age (OR = 0.261; 95% CI.104–0.605, P = 0.003), sex (men: OR = 3.866; 95% CI 1.758–8.88; *P* < 0.001), ETE (OR = 3.821; 95% CI 1.168–13.861; *P* = 0.032), D/T (OR = 72.411; 95% CI 5.483–1212.497; *P* < 0.001), and coexistent benign lesions (OR = 3.112; 95% CI 1.407–7.303; *P* = 0.007) were shown to be substantially related to CLNM in cN0 unilateral PTMC (Table 2).

**Table 1:** Univariate logistic regression of predicting cervical lymph node metastasis in papillary thyroid microcarcinoma (PTMC) patients.

**Variables**	**OR**	**95% CI**		***P*-value**
		**Lower**	**Upper**	
**Age**				
<55	1.000			
≥55	0.352	0.160	0.726	**0.006**
**Sex**				
Female	1.000			
Male	2.405	1.249	4.665	**<0.001**
**Tumor size (mm)**	1.253	1.055	1.499	**0.012**
**Tumor invasion**				
Localized	1.000			
Capsular involvement	1.708	0.879	3.366	0.117
Extrathyroidal extension	5.619	1.934	17.966	**<0.001**
**Multifocality**				
No	1.000			
Yes	1.238	0.461	3.173	0.660
**Thyroid echo**				
Homogeneous	1.000			
Heterogeneous	1.306	0.349	6.226	0.706
**Lobe thickness (mm)**	0.951	0.849	1.059	0.366
**D/T**	19.985	2.391	186.932	**0.007**
**Shape**				
Regular	1.000			
Irregular	1.049	0.447	2.609	0.914
**Microcalcification**				
Negative	1.000			
Positive	1.359	0.733	2.546	0.333
**Aspect ratio**				
≤ 1	1.000			
>1	0.702	0.371	1.334	0.277
**Blood flow**				
None	1.000			
Surrounding	1.109	0.556	2.211	0.767
Internal	1.642	0.713	3.768	0.240
**Location**				
Upper	1.000			
Middle	1.648	0.591	5.371	0.366
Lower	2.782	0.959	9.350	**0.073**
**Coexistent with benign lesions**				
No	1.000			
Yes	1.817	0.943	3.608	**0.080**

*The bold values mean significant differences*.

**Table 2:** Multivariate logistic regression of predicting cervical lymph node metastasis in PTMC patients.

**Variables**	**OR**	**95%CI**		***P*-value**
		**Lower**	**Upper**	
**Age**				
<55	1.000			
≥55	0.261	0.104	0.605	**0.003**
**Sex**				
Female	1.000			
Male	3.866	1.758	8.880	**<0.001**
**Tumor invasion**				
Localized	1.000			
Capsular involvement	1.062	0.485	2.308	0.879
Extrathyroidal extension	3.821	1.168	13.861	**0.032**
**D/T**	72.411	5.483	1,212.497	**<0.001**
**Location**				
Upper	1.000			
Middle	1.425	0.444	5.262	0.569
Lower	2.809	0.824	10.991	0.113
**Coexistent with benign lesions**				
No	1.000			
Yes	3.112	1.407	7.303	**0.007**

*The bold values mean significant differences*.

### Nomogram Construction

**Figure 3:**
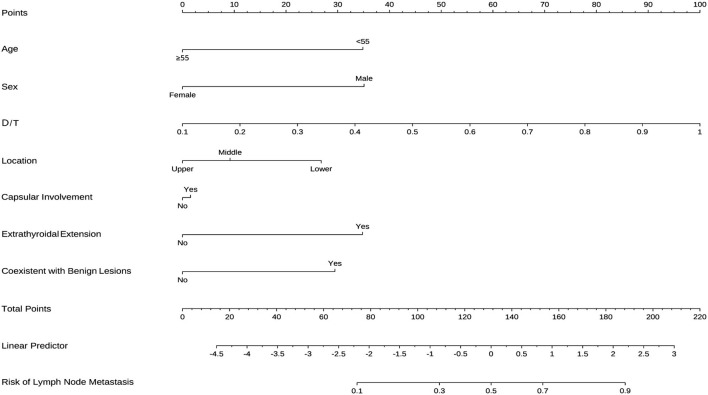
Nomogram for predicting CLNM in cN0 unilateral PTMC patients.

**Figure 4:**
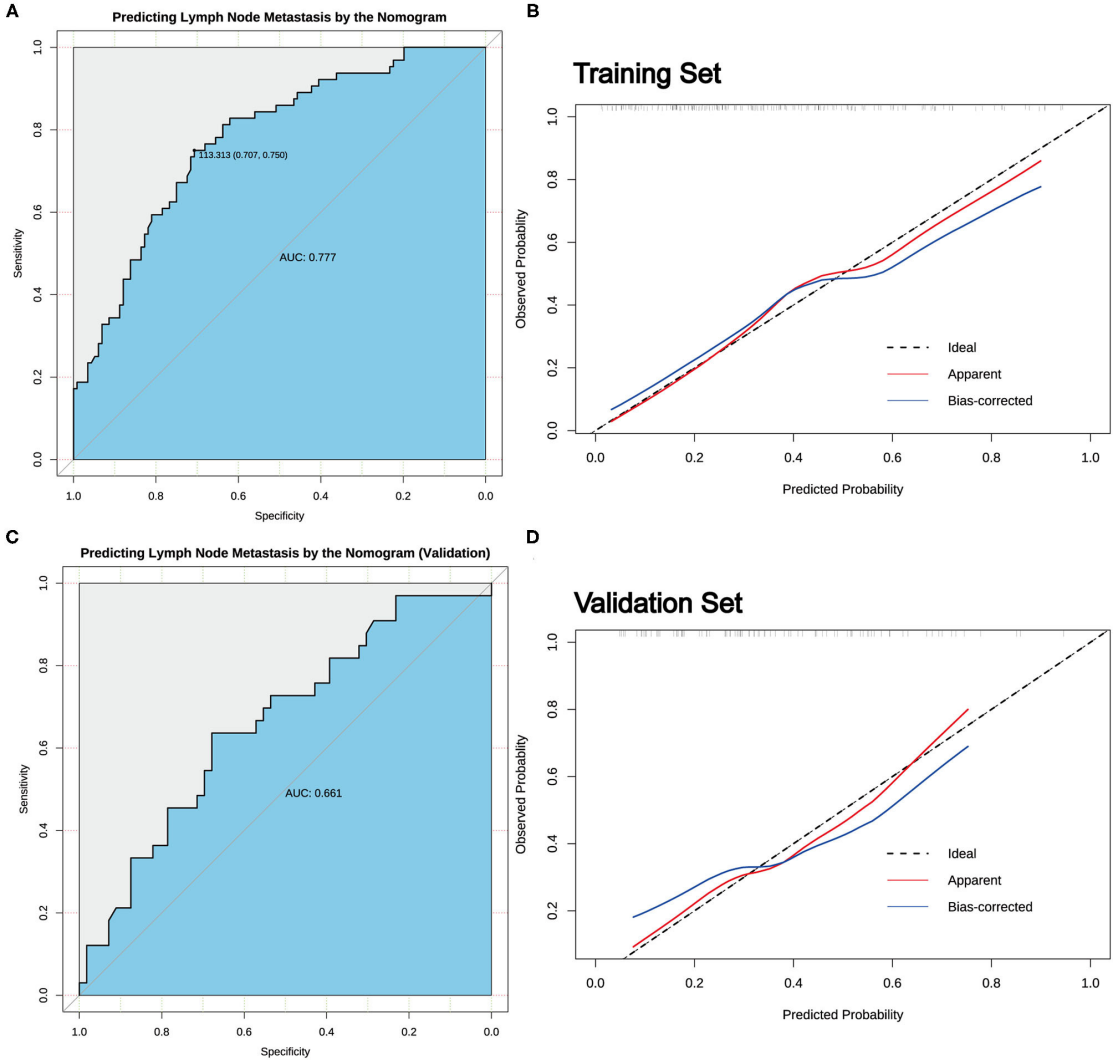
The predictive value of the nomogram. **(A)** The receiver operating characteristic (ROC) curve for predicting CLNM in cN0 unilateral PTMC patients in the training set. **(B)** Calibration curves of the nomogram for predicting CLNM in cN0 unilateral PTMC patients in the training set. **(C)** The ROC curve for predicting CLNM in cN0 unilateral PTMC patients in the validation set. **(D)** Calibration curves of the nomogram for predicting CLNM in cN0 unilateral PTMC patients of the validation set.

According to the result of LASSO regression analysis, a nomogram for predicting CLNM in patients with cN0 unilateral PTMC based on the seven factors with non-zero coefficients, including age, sex, tumor size, the presence of ETE, D/T, tumor location, and coexistent benign lesions, was constructed (Figure 3). It is worth mentioning that D/T yielded the largest contribution to the prediction model, while capsular involvement provided the next largest contribution. The predictive value of the nomogram was verified by ROC curves (AUC =0.777; 95% CI.708–0.847; Figure 4), in which the optimal cutoff score was 113.313 with a sensitivity and specificity of 0.75 and 0.707, respectively. The nomogram exhibited an excellent consistency, which was evaluated by the calibration curve (Figure 4). Moreover, the predictive value of the nomogram was also evaluated in the validation set. The AUC value was 0.661 (95% CI.544–0.778; Figure 4), and the calibration curve also showed a fairly good consistency (Figure 4).

## Discussion

With the development of HUS and FNA, PTMC now accounts for the majority of PTC diagnoses (15). PTMC with neither local invasion/distant metastasis nor convincing cytological evidence could be defined as low-risk PTMC. The 2015 ATA management guidelines recommend that active surveillance (AS) can be taken instead of surgical treatment for low-risk PTMC to avoid overdiagnosis and overtreatment (1). However, the study of Choi et al. (16) put forward a point of view that, due to clinically apparent lymph node metastasis, AS rarely leads to favorable oncological outcomes. It is highly likely that lymph node metastasis will eventually lead to local recurrence and secondary operations, which greatly increase the risk of hypoparathyroidism and RLN injury during surgery and reduce the quality of life of the patient (17, 18). Therefore, it is necessary to precisely stratify patients with PTMC and evaluate the risk of CLNM in these patients; hence, several researchers have attempted to develop optimal predictive models. Unfortunately, previous models for predicting CLNM in patients with cN0 PTMC have been mostly based on postoperatively obtained pathological features, such as ETE, tumor size, and multifocality (19, 20). For preoperative evaluation, Sun et al. (13) developed a nomogram based on US features and clinical characteristics for the preoperative diagnosis of CLNM in patients with PTMC, which included male sex, age <45 years, multifocality, tumor size, ETE, microcalcification, and the detection of suspicious metastatic lymph nodes. However, patients with suspicious positive US lymph nodes are supposed to receive CLND, and thus the rationality of the inclusion of indicators remains controversial. In addition, a lack of further validation of the results has limited their accuracy and credibility. As a result, a noninvasive and preoperative evaluation method has been developed in our present research to assess CLNM in patients with PTMC.

Therefore, we established a nomogram based on clinical and preoperative US imaging characteristics in order to predict the probability of CLNM in patients with PTMC. The nomogram, which included age, sex, tumor size, presence of ETE, D/T, tumor location, and coexistent benign lesions, and the model exhibited an excellent predictive value with an AUC as high as 0.777. In our nomogram, D/T is a novel indicator, and no other research has proposed a similar indicator. The relationship of tumor size with CLNM is still under debate. Siddiqui et al. (21) reported that tumor size was not correlated with central lymph node involvement. However, the study of Zheng et al. (22) reported increasing tumor size (≥5 mm) to be related to a significantly higher risk of PTMC central lymph node involvement, which is consistent with most studies (23, 24). We considered that the reason for these different results may be attributed to the thickness of the thyroid gland. It is worth mentioning that the use of D/T can eliminate the interference of different ipsilateral thyroid lobe thicknesses to reflect the true effect of tumor size. In addition, the evaluation of ETE in US imaging is reliable. Although ETE is a type of clinicopathological factor associated with CLNM (25, 26), the ETE preoperative evaluation by the US has been shown to be satisfactory in some studies (27, 28).

According to our results, the nomogram could be employed to predict the existence of CLNM in order to determine a personal surgical volume and therapeutic strategy which might avoid overtreatment or a missed diagnosis of CLNM. Hence, for patients with cN0 unilateral PTMC diagnosed by US imaging, when the score calculated by nomogram is ≥ 113.313, patients can be identified as high-risk PTMC patients. Prophylactic CLND under proper protection of the parathyroid and RLN is suggested in these patients to avoid reoperations and recurrence. For patients identified as low-risk PTMC patients, prophylactic CLND might be spared due to the fairly low risk of CLNM and the potential for surgical complications to occur during CLND. Meanwhile, follow-up of patients after the operation is certainly important, especially for high-risk patients. It is also crucial to note that this cutoff value was only used as a reference to illustrate our approach of applying the nomogram during clinical practice. A more optimal nomogram and cutoff value should be developed and determined based on further research with larger sample size.

Our study was limited in that it was a single-center, retrospective study, and the sample size was relatively small. Compared with prospective studies, the errors and biases in retrospective studies are often higher. Moreover, the evaluation of US imaging factors involves some subjectivity. Nonetheless, our study showed excellent interobserver agreement. Therefore, a multicenter study with a larger number of cases is necessary to minimize selection bias and verify these findings.

In conclusion, we developed a nomogram based on clinical and US imaging characteristics that included novel indicators to predict the probability of CLNM in patients with PTMC. Further prospective research to observe oncological outcomes is necessary to determine whether the nomogram could guide a personalized surgical volume and surgical approach.

## Data Availability Statement

The data involves patient privacy and cannot be openly disclosed, further reasonable requests can be made to the corresponding author/s.

## Ethics Statement

The studies involving human participants were reviewed and approved by Xuanwu Hospital, Capital Medical University based in Beijing, China {20 April 2020, ID [2020]055}. The patients/participants provided their written informed consent to participate in this study.

## Author Contributions

HK: conception and design and administrative support. KL, JZ, and HK: provision of study materials or patients. LZ, YL, and YZ: collection and assembly of data. YL: data analysis and interpretation. All authors final approval of manuscript and manuscript writing.

## Funding

This work was supported by the Beijing Municipal Health System Academic Leaders of High-level Health Personnel Program, China(2011-2-28).

## Conflict of Interest

The authors declare that the research was conducted in the absence of any commercial or financial relationships that could be construed as a potential conflict of interest.

## Publisher's Note

All claims expressed in this article are solely those of the authors and do not necessarily represent those of their affiliated organizations, or those of the publisher, the editors and the reviewers. Any product that may be evaluated in this article, or claim that may be made by its manufacturer, is not guaranteed or endorsed by the publisher.
